# Global distribution and drivers of language extinction risk

**DOI:** 10.1098/rspb.2014.1574

**Published:** 2014-10-22

**Authors:** Tatsuya Amano, Brody Sandel, Heidi Eager, Edouard Bulteau, Jens-Christian Svenning, Bo Dalsgaard, Carsten Rahbek, Richard G. Davies, William J. Sutherland

**Affiliations:** 1 Conservation Science Group, Department of Zoology, University of Cambridge, Cambridge CB2 3EJ, UK; 2 Section for Ecoinformatics and Biodiversity, Department of Bioscience, Aarhus University, Aarhus 8000 C, Denmark; 3 Research Laboratory for Archaeology and the History of Art, School of Archaeology, University of Oxford, Oxford OX1 2HU, UK; 4 Department of Ecology and Evolutionary Biology, Cornell University, Corson Hall, Ithaca, NY 14853-2701, USA; 5 Ecole Polytechnique, Route de Saclay, 91120 Palaiseau, France; 6 Center for Macroecology, Evolution, and Climate, Natural History Museum of Denmark, University of Copenhagen, Universitetsparken 15, 2100 Copenhagen, Denmark; 7 School of Biological Sciences, University of East Anglia, Norwich NR4 7TJ, UK

**Keywords:** cultural conservation, cultural diversity, endangered languages, human ecology, language conservation, macroecology

## Abstract

Many of the world's languages face serious risk of extinction. Efforts to prevent this cultural loss are severely constrained by a poor understanding of the geographical patterns and drivers of extinction risk. We quantify the global distribution of language extinction risk—represented by small range and speaker population sizes and rapid declines in the number of speakers—and identify the underlying environmental and socioeconomic drivers. We show that both small range and speaker population sizes are associated with rapid declines in speaker numbers, causing 25% of existing languages to be threatened based on criteria used for species. Language range and population sizes are small in tropical and arctic regions, particularly in areas with high rainfall, high topographic heterogeneity and/or rapidly growing human populations. By contrast, recent speaker declines have mainly occurred at high latitudes and are strongly linked to high economic growth. Threatened languages are numerous in the tropics, the Himalayas and northwestern North America. These results indicate that small-population languages remaining in economically developed regions are seriously threatened by continued speaker declines. However, risks of future language losses are especially high in the tropics and in the Himalayas, as these regions harbour many small-population languages and are undergoing rapid economic growth.

## 1. Introduction

Languages are now rapidly being lost [1–3] at a rate of extinction exceeding the well-known catastrophic loss of biodiversity [4]. Serious concerns over the impending loss of human cultural diversity [1] have driven several international organizations, such as the United Nations Educational, Scientific, and Cultural Organization, the Convention on Biological Diversity, the World Wide Fund for Nature and the International Union for Conservation of Nature (IUCN), to actively engage in the conservation of linguistic diversity [5–8]. Earlier studies have reported the distribution of language diversity and its congruence with species diversity [9–11], identified areas with a high number of endangered languages [12], and tested for factors affecting range size in Old World languages [13] and per-country linguistic persistence globally [14]. Numerous schemes have also been proposed to categorize levels of language endangerment [2,15,16] and a range of processes have been listed as causes of language endangerment (e.g. [3,17–19]), most notably globalization and modernization [3,19]. However, few studies to date have assessed the relative roles of different drivers in explaining the geographical distribution of language extinction risk, limiting the knowledge-base for efforts to prevent this cultural loss [20]. For example, although globalization has been regarded as an important factor behind language endangerment, there has been little research effort worldwide to quantify the overall impact of globalization on endangered languages [3,19].

We address this knowledge gap by evaluating language extinction risk represented by small geographical range sizes, small speaker population sizes and rapid declines in speaker numbers. These three risk components are selected based on the IUCN Red List criteria, which have been established to evaluate the extinction risk of species, i.e. symptoms of endangerment rather than causes [21,22], but are also expected to effectively represent the extinction risk of languages. Rapid declines in the number of speakers, which can be caused by both language shifts and speaker deaths, clearly represent higher extinction risk, since they can potentially swamp any influence of population size on the time to extinction [21]. Small range and speaker population sizes can lead to high extinction risk due to the effect of demographic and environmental stochasticity on speaker population dynamics [21] as well as reduced competitive abilities [12], all of which are known as important processes causing language extinction (e.g. [17,23–25]). Theoretical studies have also shown that the range and speaker population sizes of a language are key factors for explaining the time to extinction [26–28]. These risk components have also been used in other schemes to categorize levels of language endangerment (see the electronic supplementary material, appendix A for more detail). The advantage of focusing on these three risk components is that data are available for the whole spectrum of languages, from endangered to least-threatened languages. This allows us to effectively test the role of potential drivers in shaping the different levels of language endangerment.

We first collect information on the range size, speaker population size and speaker growth rate (i.e. changes in the number of speakers) of the world's languages, and assess interrelations among these three risk components to understand how they contribute to shaping extinction risk in languages. We then quantify the geographical distribution of these risk components and identify the underlying drivers by linking these geographical patterns to potentially important environmental and socioeconomic factors. In doing so, we test two hypotheses. The first hypothesis predicts that range size and speaker population size should be strongly associated with environmental factors, reflecting their historical influence on language evolution and persistence [10,29]. By contrast, reflecting the accelerating and pervasive economic and cultural globalization, the second hypothesis predicts that recent speaker declines should largely be explained by current socioeconomic factors, notably economic growth and globalization [12,19,29]. As there are other criteria proposed to categorize the levels of language endangerment [18,19,30], we also conduct the same analysis for risks caused by insufficient intergenerational language transmission, which has been suggested to be an important determinant of language vitality [15]. Finally, we map the distribution of threatened languages as categorized using the IUCN criteria to identify the hotspots of language extinction risk. To verify the validity of the assessment, we compare the map based on the IUCN criteria with the distribution of endangered languages listed in the Catalogue of Endangered Languages [16], another global assessment using a different set of criteria. We further test for spatial discrepancy between threatened languages and past language extinctions to assess the idea that languages may appear less threatened in regions where many languages have already become extinct [31]. Our results constitute an important step for understanding the processes that drive language extinctions and for developing and prioritizing future linguistic conservation efforts in terms of interventions, areas and languages [10]. Our study also contributes to a basic understanding of the origin and maintenance of human cultural diversity [11], just as ecological studies have focused on the distribution of species range size to understand the determinants of species diversity [32].

## 2. Material and methods

### (a) Data

The range size and speaker population size of each language were estimated based on information from the Ethnologue, 16th edition [25], which represents the most authoritative and only globally comprehensive source of basic information about languages and their speakers. The data were assembled in a Geographical Information System by Global Mapping International as the WLMS database [33], providing georeferenced polygons showing their geographical range, associated with information on speaker population size. Languages that are given as points or have no known location/population size were excluded, leaving 6359 (92% of the known 6909 languages) and 6569 (95%) languages in the analysis for range size and population size, respectively. The total area (km^2^) of all the polygons for each language was defined as the range size, and the latest estimate of the total number of mother-tongue speakers in the polygon attributes as the speaker population size.

Speaker growth rates were estimated using the index of linguistic diversity (ILD) database [34], updated with the Ethnologue, 16th edition; this database provides information on temporal changes in the speaker population size (i.e. estimates of speaker population size and survey years) between 1949 and 2005 for 1500 languages selected at random from the Ethnologue. The ILD database is currently the only global database with information on changes in the population size of languages. To estimate speaker growth rate, we selected languages with at least three records of speaker population size, including at least one non-zero record. This resulted in 649 languages, including 24 languages that have become extinct after 1949, to be analysed for their speaker growth rate. This sample size represents approximately 9% of all known languages but the languages included are well scattered across the globe, roughly following the pattern of distribution for all the languages (see electronic supplementary material, figures S1 and S2). The biases in range size and speaker population size between the 649 languages and all available languages in the ILD and WLMS databases were also very small (see the electronic supplementary material, figure S3, for more detail). Thus, we expect the effect of using the sample of 649 languages for drawing conclusions to be minimal. The level of intergenerational transmission in each language was derived from the Atlas of the World's languages in danger [15] (see the electronic supplementary material, appendix B for more detail).

Data on potential drivers of extinction risk were derived from different global data sources (electronic supplementary material, appendix C). Since records used for estimating speaker growth rates were mostly collected between 1978 and 2000 (see the electronic supplementary material, figure S4), we used data sources from this period as much as possible. Though information on gross domestic product (GDP) and globalization was only available at the country level, the obtained data fit the purpose of this analysis, given that the economic status and degree of globalization of a country, not of each speaker, are expected to cause language shifts through educational developments [19] and the economic benefits of speaking national and global languages [17]. Language richness in each cell was defined as the total number of languages whose geographical range overlaps that cell, based on the WLMS database. The land area of a latitudinal band was calculated as the sum of the land area of all grid cells within the same latitude at the 2° resolution.

### (b) Analysis

For the 649 languages with more than two records, the speaker growth rate was estimated by fitting a generalized linear model (Poisson distribution and log link) with speaker population size as the response variable and year as the explanatory variable. The estimated coefficient for the year term was defined as the speaker growth rate of each language.

We investigated the bivariate relationships among range size, speaker population size and speaker growth rate by comparing the Akaike information criterion (AIC) of four different models [35]: null, linear, quadratic and segmented regression models. We used the R package ‘segmented’ [36] to implement the segmented regression. Note that the initial population size (i.e. the record of speaker population size in the oldest survey year) was used in the analysis of the relationship between speaker growth rate and population size, in order to avoid circularity. However, since the oldest survey year varies among languages, the relationship between initial population size and speaker growth rate can be circular if the oldest survey year tends to be later in declining languages. But the validity of this approach was supported, as there was no significant correlation between speaker growth rate and oldest survey years (Kendall's *τ* = −0.040, *p* = 0.139).

To identify factors associated with extinction risk, we first projected the language range map onto a Behrmann equal-area cylindrical projection and converted the shape files to grid cells with a grain size of 192.9725 km, or approximately 2° at 30° N/S. A grid cell was considered to contain a language if its range polygon covered any portion of the grid cell. We then calculated the median range size, population size and speaker growth rate of all languages within each cell. The median risk due to insufficient intergenerational transmission across all languages within each cell was also calculated, assuming vulnerable = 1, definitely endangered = 2, severely endangered = 3, critically endangered = 4 and all others = 0 based on [15]. We also aggregated all the explanatory variables to the same 2° × 2° grid cells and calculated mean values in each cell. We excluded grid cells containing less than 50% land area or those lacking any languages with data on each response variable, resulting in a global dataset of 3409 grid cells for range size and speaker population size, 1811 for speaker growth rate and 3408 for intergenerational transmission. To explore latitudinal gradients in range size, speaker population size and speaker growth rate, we calculated the median values of all grid cells in the same latitudinal band.

We developed hypotheses for the effects of a suite of environmental and socioeconomic factors on language extinction risk (see the electronic supplementary material, table S1). Owing to high correlations (|*r*| > 0.8) with either temperature seasonality or GDP per capita (see the electronic supplementary material, table S2), annual mean temperature, globalization index and land area within the same latitudinal band were excluded from the analysis, so only 10 variables were used. All tolerance values for the remaining 10 variables exceeded 0.31, indicating sufficient independence of the explanatory variables.

In the analyses for determining the extent to which each factor was associated with language extinction risk, the response variables were log_10_ (median range size), log_10_ (median speaker population size), median speaker growth rate and median risk due to insufficient intergenerational transmission, and the explanatory variables were annual precipitation, vegetation productivity, temperature seasonality, precipitation seasonality, elevation range, habitat diversity, mean population density, mean population change, mean GDP per capita and language richness. We did not use language richness as an explanatory variable in the analyses for range size and speaker population size because high language richness can also be a consequence of small range size and speaker population size, making it difficult to infer their causal relationships. We first tested the association between the response variables and explanatory variables with the non-spatial ordinary least-squares (OLS) models. The OLS models suffered from strong spatial autocorrelation in model residuals, based on Moran's *I* estimated with the package ‘ncf’ [37] in R (see the electronic supplementary material, figures S5). Thus, we decided to adopt simultaneous autoregressive (SAR) error models in all the analyses. SAR error models were first fitted using a range of neighbourhood distances (from 250 to 500 km in 50-km intervals for all four variables as well as 1000 and 1500 km for speaker growth rate and intergenerational transmission and 1000 and 2000 km for range and population sizes). Distances of 450, 350, 300 and 350 km, which showed the smallest AIC, were adopted in the following analysis for range size, speaker population size, speaker growth rate and intergenerational transmission, respectively. The SAR error models successfully removed the spatial autocorrelation in the model residuals (electronic supplementary material, figures S5). To account for model selection uncertainty, we adopted a multi-model inference approach [35]. We first generated a set of models with all possible parameter subsets, which were then fitted to the data using the SAR error models and ranked by ΔAIC values. We calculated Akaike weights (*w_i_*) for each model as an indicator of relative support and summed these across the candidate set to find the 95%-confidence set [35]. Model-averaged coefficients, standard errors and *z*-values (weighted by *w_i_*) were also calculated across the 95% set. The sum of *w_i_* of models including each variable (Σ *w_i_*) and model-averaged *z*-values were used as indicators of parameter importance across models. All analyses were conducted in R 2.15.0 [38]; the SAR models were fitted with the row-standardized (‘*W*’) coding using the package ‘spdep’ [39], and model averaging was conducted using the package ‘MuMIn’ [40]. Considering the argument that a particular spatial model cannot always be assumed to be more correct than non-spatial models [41], we also provided results based on model averaging of OLS models.

### (c) Categorization of threatened languages

We used the IUCN criteria [22] to evaluate if a language belonged to any of the three threatened categories: Critically Endangered, Endangered and Vulnerable (see the electronic supplementary material, table S3). Note that each of the five criteria uses different combinations of the three aspects of extinction risk: A3, D1 and D2 are based on population declines, population size and range size, respectively, while the other two criteria are based on the combination of population declines with range size (B1) and population declines with population size (C1). Using the same 2° × 2° grid cells, we mapped the number of threatened languages (i.e. all languages categorized as Critically Endangered, Endangered and Vulnerable) based on each of the five criteria. We also mapped the number of endangered languages listed in the Catalogue of Endangered Languages [16] using the same grid cells (see the electronic supplementary material, appendix B for more details).

We also calculated the number of extinct languages in each cell based on the location of the last known population of speakers of extinct languages, derived from the Extinct Language point features in the WLMS database.

## 3. Results

### (a) Extinction risk of each language

**Figure 1.**
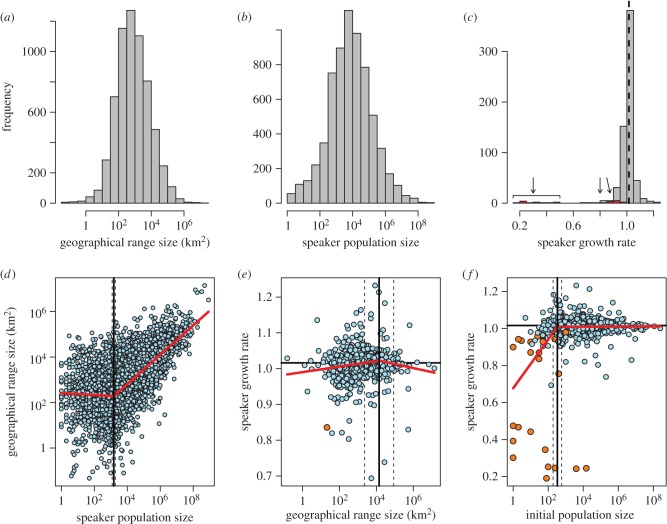
Histograms of (*a*) language range size (km^2^, *n* = 6359), (*b*) language population size (*n* = 6569), (*c*) speaker growth rate (*n* = 649) and (*d*–*f*) their pairwise relationships. The black vertical line in (*c*) and horizontal lines in (*e*) and (*f*) indicate the mean human population growth rate between 1980 and 2000, and red bars (shown with arrows) in (*c*) and orange circles in (*e*) and (*f*) show languages that have become extinct after 1949. Red lines in (*d*)–(*f*) show the fitted segmented regression, and vertical solid and dashed lines are the estimated thresholds and their 95% confidence intervals, respectively (see Material and methods and electronic supplementary material, table S4 for more detail).

The frequency of range size and speaker population size approximated a lognormal distribution (figure 1*a*,*b*), indicating that there are a huge number of small-range and small-population languages. Based on the IUCN's criteria (see the electronic supplementary material, table S3), a range size smaller than 20 km^2^ causes species to be defined as threatened, assuming that the population is prone to serious threats within a short time period, and 291 languages meet this criterion. Additionally, 1496 languages have a speaker population size smaller than 1000, another of IUCN's thresholds for defining species as threatened (electronic supplementary material, table S3). The frequency distribution of speaker growth rate centred on the mean growth rate of global human population (1.016 between 1980 and 2000) with a long left tail, indicating the presence of severely declining languages (figure 1*c*). In total, 193 (29.7%) of the 649 languages showed a recent decline in the number of speakers, and in 168 (25.9%) languages the estimated rate of decline exceeded 30% over three generations, causing these languages to be defined as threatened under the IUCN's criteria (electronic supplementary material, table S3). Consequently, 1705 (24.7%) of the known 6909 languages fulfil IUCN's criteria for being defined as threatened because of a small range size, small speaker population size and rapid speaker declines.

All three pairwise relationships among range size, speaker population size and speaker growth rate were better expressed by a segmented linear relationship with a threshold than with the null, linear or quadratic models based on AIC (figure 1*d–f*; see the electronic supplementary material, table S4). Range size was unrelated to speaker population size below a certain level (estimated mean threshold: 1455, 95% CI: 1160–1826), after which range size steeply increased with population size (figure 1*d*). The absence of a range-population size correlation at low speaker population sizes may be because in some regions, such as deserts, even small numbers of speakers can occupy large territories. Speaker growth rate and range size were essentially unrelated, but with a weak positive association below the threshold range size (mean: 15 155 km^2^, 95% CI: 2415–95 118 km^2^; figure 1*e*).

On the other hand, there was a clear difference in speaker growth rate between languages below and above the threshold speaker population size. Above the threshold (mean: 334, 95% CI: 191–587), many languages have survived successfully with speaker growth rates similar to the mean growth rate of the global human population, whereas languages with speaker population sizes below the threshold have shown severe declines and, in many cases, have consequently become extinct in recent years (figure 1*f*).

### (b) Distribution and drivers of extinction risk

**Figure 2.**
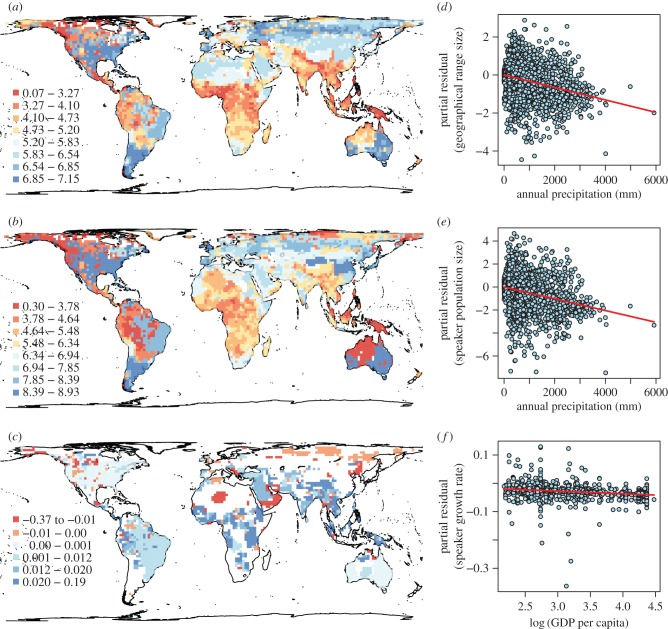
Global maps of extinction-risk components for languages and the important underlying drivers. The maps show median (*a*) language range size (km^2^), (*b*) language population size and (*c*) speaker growth rate. Medians were calculated for log_10_-transformed range size and population size and for speaker growth rate. The plots show the individual effects of (*d*) annual precipitation on language range size, (*e*) annual precipitation on language population size and (*f*) GDP per capita on speaker growth rate, after effects of other variables and spatial autocorrelation have been partialled out. Variables shown here are one of the most important variables in each of the best SAR error models (i.e. those with the smallest AIC); lines represent regression lines based on coefficients estimated in the best models. Other important variables are shown in the electronic supplementary material, figures S8–10.

Both range size and speaker population size were generally small in both the tropics and the Arctic region (figure 2*a*,*b*), and there was a tendency for both to increase from low to high latitudes, but to decrease above 60°N (see the electronic supplementary material, figure S6*a*,*b*). Speaker growth rates tended to be lower at higher latitudes (electronic supplementary material, figure S6*c*), with particularly marked speaker declines in North America, Europe, Russia, Australia and the desert areas in Africa and the Middle East (figure 2*c*). Median speaker growth rates were generally positive close to the equator (electronic supplementary material, figure S6*c*). The risk due to insufficient language intergenerational transmission showed a similar geographical pattern to speaker growth rate, being particularly high in North America, northern Eurasia, a part of Australia and South America and desert areas in Africa (electronic supplementary material, figure S7).

Model averaging across SAR models with varying sets of explanatory variables supported the hypothesis that environmental factors play an important role in determining language range and population size. Small range sizes were associated particularly with high precipitation, vegetation productivity, topographic heterogeneity and habitat heterogeneity (table 1 and figure 2*d*; also see the electronic supplementary material, figure S8*a*–*c*). Similarly, small speaker population sizes were associated with high precipitation and topographic heterogeneity (table 1 and figure 2*e*; electronic supplementary material, figure S9*a*). Nevertheless, some socioeconomic factors were also important in explaining these extinction-risk components, such as low GDP per capita for small range sizes, low human population density for small speaker population sizes, and rapid human population growth for both (table 1; electronic supplementary material, figures S8*d*–*e* and S9*b*–*c*).

**Table 1.** Model-averaged results based on SAR error models for the effect of environmental and socioeconomic factors on language range size, population size and speaker growth rate. Language richness was not included in the models for range size and population size (see Material and methods for more detail). Coefficients, their SEs and *z-*values show weighted-average values across 95% sets of SAR error models with different parameter combinations by Akaike weights (*w_i_*). Σ *w_i_* for each variable shows the sum of *w_i_* of models including the variable, reflecting the relative importance of each variable. Results with *z-*values > 2.0 are shown in bold. Nagelkerke pseudo-*R^2^* for the full model was 0.60, 0.50 and 0.73 for range size, population size and speaker growth rate, respectively.

explanatory variables	geographical range size				population size				speaker growth rate			
	coefficient	s.e.	*z*-value	Σ *w_i_*	coefficient	s.e.	*z*-value	Σ *w_i_*	coefficient	s.e.	*z*-value	Σ *w_i_*
environmental factors												
annual precipitation	−**3.30 × 10^−4^**	**0.58 × 10^−4^**	**5.66**	**1.00**	−**5.14 × 10^−4^**	**0.88 × 10^−4^**	**5.83**	**1.00**	2.16 × 10^−6^	2.26 × 10^−6^	0.96	0.38
vegetation productivity	**−4.35 × 10^−3^**	**1.79 × 10^−3^**	**2.44**	**0.89**	−2.83 × 10^−3^	2.92 × 10^−3^	0.97	0.37	5.88 × 10^−5^	6.63 × 10^−5^	0.89	0.35
temperature seasonality	1.22 × 10^−5^	1.69 × 10^−5^	0.72	0.32	−1.18 × 10^−5^	2.09 × 10^−5^	0.56	0.29	**−1.17 × 10^−6^**	**0.57 × 10^−6^**	**2.05**	**0.75**
precipitation seasonality	0.47 × 10^−3^	1.21 × 10^−3^	0.39	0.28	−2.65 × 10^−3^	1.90 × 10^−3^	1.40	0.49	2.84 × 10^−5^	5.28 × 10^−5^	0.54	0.29
elevation range	**−9.84 × 10^−5^**	**2.23 × 10^−5^**	**4.42**	**1.00**	**−9.63 × 10^−5^**	**3.69 × 10^−5^**	**2.61**	**0.95**	4.50 × 10^−7^	8.64 × 10^−7^	0.52	0.29
habitat diversity	−**0.15**	**0.05**	**3.07**	**0.99**	−0.11	0.09	1.26	0.46	−1.21 × 10^−3^	1.84 × 10^−3^	0.66	0.30
socioeconomic factors												
mean population density	0.93 × 10^−2^	2.88 × 10^−2^	0.32	0.27	**0.28**	**0.04**	**6.32**	**1.00**	−0.66 × 10^−4^	1.10 × 10^−3^	0.60	0.30
population change	−**0.11**	**0.06**	**2.03**	**0.73**	**−0.28**	**0.08**	**3.46**	**1.00**	1.07 × 10^−3^	2.43 × 10^−3^	0.44	0.29
GDP per capita	**0.28**	**0.09**	**3.13**	**0.99**	0.03	0.12	0.253	0.27	**−9.58 × 10^−3^**	**3.51 × 10^−3^**	**2.73**	**0.97**
language richness									−3.03 × 10^−3^	2.64 × 10^−3^	1.15	0.25

By contrast, the most important factor for explaining speaker growth rate was a socioeconomic factor, GDP per capita, followed by temperature seasonality with less importance (table 1). Languages have recently declined particularly in areas with high GDP per capita (figure 2*f*) and temperature seasonality (electronic supplementary material, figure S10). The result was similar in language intergenerational transmission, where the global geographical variation was mostly explained by socioeconomic factors, including GDP per capita (electronic supplementary material, table S5). The risk due to insufficient intergenerational transmission was particularly high in areas with high GDP per capita and temperature seasonality as well as in those with low mean population density and high numbers of languages (electronic supplementary material, table S5 and figure S11).

The results were largely similar when based on OLS regression models (see the electronic supplementary material, table S6). However, the relative importance changed slightly in some factors (e.g. seasonality, habitat diversity and population density as well as GDP per capita were similarly important for speaker growth rate) and, in a rare case, the direction of the effect also changed (e.g. vegetation productivity for range size). This supports, at least qualitatively, the conclusions based on the SAR models.

### (c) Distribution of threatened and extinct languages

**Figure 3.**
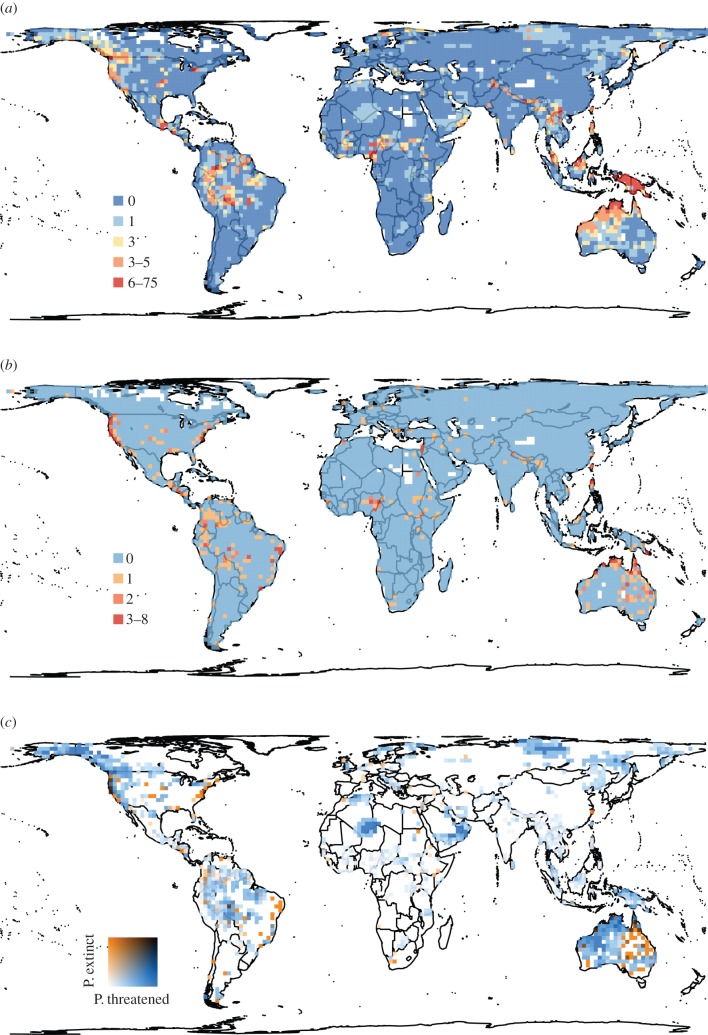
Global maps of (*a*) threatened language richness based on the IUCN criteria, (*b*) extinct language richness and (*c*) relationships between the proportion of threatened to total extant language richness and the proportion of extinct to total extant language richness. Note that the number of extinct languages in each cell is based on the location of the last known population of speakers of extinct languages. In (*c*), blue areas, which have disproportionately large numbers of threatened languages and have experienced few extinction events, are of particular conservation concern.

Hotspots of threatened language richness were particularly evident in the tropics, the Himalayas, northern Australia, eastern Eurasia and northern Russia/Scandinavia, and northwestern North America (figure 3*a*). These areas are characterized by high rainfall, high topographic heterogeneity and/or rapidly growing human populations (see the electronic supplementary material, figure S12), and encompass many languages that are threatened because of their small speaker population sizes (electronic supplementary material, figure S13*b*). The distribution of threatened language richness corresponded approximately to that of total language richness (electronic supplementary material, figure S1*a*). However, northwestern Australia, New Guinea, northern Eurasia, desert areas in Africa and the Middle East, Brazil and northwestern North America had disproportionately large numbers of threatened languages and have so far experienced few extinctions (figure 3*c*). This indicates high current threat levels, at least partly due to high economic growth or temperature seasonality in these regions. On the other hand, a few major languages are now dominant on the east coasts of the Americas and Australia (figure 2*a*,*b*; electronic supplementary material, figure S1*a*), with most other languages having already gone extinct (figure 3*c*). This supports the extinction filter hypothesis [31] that mainly large-range languages remain in regions where many languages have already become extinct, thereby causing these areas to appear less prone to language losses, as has been observed in mammals [42].

Threatened languages based on the Catalogue of Endangered Languages showed a similar spatial pattern to that based on the IUCN criteria (electronic supplementary material, figures S14 and S15). Identifiable hotspots of threatened languages are essentially the same although each hotspot was spatially generally larger when based on the Catalogue of Endangered Languages (electronic supplementary material, figure S14).

## 4. Discussion

Our results reveal how the extinction risk of human languages is formed and geographically distributed through the impact of both environmental and socioeconomic drivers across the globe. A large number of languages are now spoken in a limited area and by only a small number of people. We show that small range and speaker population sizes are both associated with rapid speaker declines, leading to a high risk of extinction. This underlines the effectiveness of the three risk components for assessing language extinction risk. In particular, severe declines and subsequent extinction in languages with speaker population sizes below about 330 indicate that the estimated threshold can be defined practically as the minimum viable population size [43] in human languages. This finding points to the presence of an Allee effect [43] (i.e. benefits from the presence of conspecifics, or in this case speakers of the same language) in human languages, potentially because of small speaker numbers being associated with both adverse language policies and voluntary language shifts [17,19], the loss of social facilitation for learning and preserving languages, reduced competitive abilities [12,24] and increased vulnerability to stochastic events [17].

The three risk components (range size, speaker population size and speaker growth rate) show striking geographical patterns at a global scale. Although small range sizes at low latitudes are a common pattern observed both in languages and species [44–46], language range size is also small in the Arctic region. This does not support a linguistic analogy of Rapoport's rule, which describes a simple increase in species range size from low to high latitudes due to increased ecological generalization [47]. The large range sizes at high southern latitudes due to the domination by European colonial languages also differ from that observed in native mammals, birds and amphibians [44–46]. On the other hand, recent language speaker declines have mainly occurred at high latitudes. In vertebrate species, populations are generally declining in the tropics, but are stable or even slightly increasing at high latitudes due in part to recent effective conservation efforts [48]. This contrast might show that linguistic conservation has been less successful and/or has attracted less attention even in economically developed temperate regions, compared to biodiversity conservation.

The geographical patterns in language extinction risk seem to be shaped by the combined effects of multiple factors. Notably, our analysis shows that both environmental and socioeconomic factors play an important role in explaining the geographical patterns in language range and population sizes. Productive and heterogeneous environments seemed to promote the evolution and/or persistence of small-range and small-population languages over thousands of years, while high human population growth apparently has the same effect, probably through an increase in potential speakers for each language. However, low human population density seems to impose a constraint on language population size at the same time. The combined effects of these environmental and socioeconomic factors can explain small language range and population sizes both at low latitudes (productive and heterogeneous environments with high human population growth) and in the Arctic regions (heterogeneous environments with low human population density).

By contrast, the dominating effect of a single socioeconomic factor, GDP per capita, on speaker growth rate suggests that economic growth and globalization (see a strong correlation between the two in the electronic supplementary material, table S2) are primary drivers of recent language speaker declines (mainly since the 1970s onwards), for instance, via associated political and educational developments and globalized socioeconomic dynamics [12,17,19]. This conclusion is also supported by the positive effect of GDP per capita on range size and many language extinctions in economically developed regions, such as the USA and Australia. That is, language speaker declines in high-GDP areas have already driven the extinction of small-ranged languages, leaving primarily large-range, major languages, as predicted by the extinction filter hypothesis [31] and also suggested for threatened bird and mammal species [42,49]. Although languages also seem to have declined in areas with high temperature seasonality, temperature seasonality was particularly high in North America and Russia, where land area within the same latitudinal band is large (Pearson's *r* between temperature seasonality and land area within the same latitudinal band = 0.841). Thus, language speaker declines in areas with high temperature seasonality may actually indicate the negative effect of the dominant English and Russian languages on other languages in these countries [2,12,17], or, more generally, that having a large land area within the same climate zone promotes the spread of dominating cultures [14]. The global distribution of risk due to insufficient intergenerational transmission was also largely explained by similar socioeconomic factors, including GDP per capita. This result, together with the small impact of human population change on speaker growth rate (table 1), supports the idea that language shifts under economic growth and globalization, rather than the loss of speaker populations themselves, represent the major underlying process of recent declines in speakers [19]. Within-country variations in the level of economic growth and globalization, though not available in this study, might further help us understand finer-scale spatial patterns in speaker growth rate.

The spatial similarity between our assessment and the Catalogue of Endangered Languages shows the effectiveness of the IUCN criteria for assessing language extinction risk. There are both advantages and disadvantages of the different sets of criteria used so far. For example, the IUCN criteria only require information that is readily available for most languages in existing databases [25,34]. The IUCN criteria use quantitative thresholds to categorize different levels of endangerments (see the electronic supplementary material, table S3), so making them less subjective, while other criteria mostly use qualitative thresholds (e.g. [18,30]). On the other hand, there are certainly fundamental differences between species and languages, such as bilingualism, language revitalization and the emergence of new languages ex nihilo [2,50,51]. The IUCN criteria may also not fully represent specific states of languages, such as domains of use and availability of written materials [18,30], which could potentially result in slight differences between our assessment and the Catalogue of Endangered Languages. Thus, we believe that the IUCN criteria and other criteria adopted in earlier schemes can be used in a complementary manner to further develop criteria for assessing language extinction risk.

We also need to be careful about the result of categorization based on the IUCN criteria related to declines in the number of speakers (i.e. A3, B1 and C1), as we could only estimate speaker growth rate for 649 languages (9% of known languages). This small sample size for speaker growth rate has inevitably led to a small number of languages being categorized as threatened due to their rapid speaker declines (see the electronic supplementary material, figure S13*c*–*e*), resulting in a small contribution of those languages to the total number of threatened languages (figure 3*a*). However, we do not consider this to be a drawback of this study, but rather believe that it reflects the lack of information on temporal population changes in human languages. The map and categorization of threatened languages can be updated readily using the approach in this study when further information becomes available in future.

Our findings highlight the contrasting status of threatened languages in hotspots within economically developed and developing regions. Economically developed regions, such as North America and Australia, have already experienced many language extinctions, most probably due to the negative impact of economic, and associated political and educational, developments [12,19]. Nevertheless, small-range and small-population languages still persist in hotspots within these regions (e.g. northwestern North America and northern Australia). Those languages need immediate attention because of their high extinction risk due to continued speaker declines and, potentially, range contractions as well. On the other hand, much of the tropics and the Himalayan region harbour many threatened languages with small range and speaker population sizes, reflecting their association with productive and topographically heterogeneous environments. As some countries in these regions are currently experiencing rapid economic growth, unless conservation efforts are targeted there, the tropics and the Himalayan region will face an elevated risk of becoming hotspots for language losses in the near future.

## Supplementary Material

ESM.revised2.Amano

## Supplementary Material

Data.Amano

## Data accessibility

All data are uploaded as the electronic supplementary material.

## References

[1] Hale K 1992 Endangered languages—on endangered languages and the safeguarding of diversity. Language 68, 1–3.

[2] Krauss M 1992 Endangered languages—the world's languages in crisis. Language 68, 4–10. (10.1353/lan.1992.0075)

[3] Austin PK Sallabank J 2011 Introduction. In The Cambridge handbook of endangered languages (eds Austin PK Sallabank J), pp. 1–24. Cambridge, UK: Cambridge University Press.

[4] Sutherland WJ 2003 Parallel extinction risk and global distribution of languages and species. Nature 423, 276–279. (10.1038/nature01607) 12748639

[5] Oviedo G Maffi L Larsen P 2000 Indigenous and traditional peoples of the world and ecoregion conservation: an integrated approach to conserving the world's biological and cultural diversity. Gland, Switzerland: WWF International—Terralingua.

[6] Convention on Biological Diversity. 2010 Decision X/2. The Strategic Plan for Biodiversity 2011–2020 and the Aichi Biodiversity Targets. Montreal, Canada: Secretariat of the Convention on Biological Diversity.

[7] UNESCO. 2005 Convention of the protection and promotion of the diversity of cultural expressions 2005. Paris, France: The United Nations Educational, Scientific and Cultural Organization.

[8] Mclvor A Fincke A Oviedo G 2008 Bio-cultural diversity and indigenous peoples journey: report from the 4th IUCN World Conservation Congress Forum, 6–9 October 2008, Barcelona, Spain.

[9] Gorenflo LJ Romaine S Mittermeier RA Walker-Painemilla K 2012 Co-occurrence of linguistic and biological diversity in biodiversity hotspots and high biodiversity wilderness areas. Proc. Natl Acad. Sci. USA 109, 8032–8037. (10.1073/pnas.1117511109) 22566626 PMC3361428

[10] Moore JL Manne L Brooks T Burgess ND Davies R Rahbek C Williams P Balmford A 2002 The distribution of cultural and biological diversity in Africa. Proc. R. Soc. Lond. B 269, 1645–1653. (10.1098/rspb.2002.2075) PMC1691086 12204124

[11] Mace R Pagel M 1995 A latitudinal gradient in the density of human languages in North America. Proc. R. Soc. Lond. B 261, 117–121. (10.1098/rspb.1995.0125)

[12] Anderson GDS 2011 Language hotspots: what (applied) linguistics and education should do about language endangerment in the twenty-first century. Lang. Educ. 25, 273–289. (10.1080/09500782.2011.577218)

[13] Currie TE Mace R 2009 Political complexity predicts the spread of ethnolinguistic groups. Proc. Natl Acad. Sci. USA 106, 7339–7344. (10.1073/pnas.0804698106) 19380740 PMC2670878

[14] Laitin DD Moortgat J Robinson AL 2012 Geographic axes and the persistence of cultural diversity. Proc. Natl Acad. Sci. USA 109, 10 263–10 268. (10.1073/pnas.1205338109) PMC3387047 22689972

[15] Moseley C 2010 Atlas of the world's languages in danger, 3rd edn Paris: UNESCO Publishing (http://www.unesco.org/culture/en/endangeredlanguages/atlas).

[16] The Linguist List at Eastern Michigan University and The University of Hawaii at Manoa. 2012 Endangered Languages: April 22, 2014. (http://www.endangeredlanguages.com)

[17] Nettle D Romaine S 2000 Vanishing voices: the extinction of the world’s languages. Oxford, UK: Oxford University Press.

[18] Brenzinger M 2003 Language vitality and endangerment. Paris: UNESCO Ad Hoc Expert Group Meeting on Endangered Languages (http://www.unesco.org/culture/en/endangeredlanguages).

[19] Grenoble LA 2011 Language ecology and endangerment. In The Cambridge handbook of endangered languages (eds Austin PK Sallabank J), pp. 27–44. Cambridge, UK: Cambridge University Press.

[20] Gavin MC 2013 Toward a mechanistic understanding of linguistic diversity. BioScience 63, 524–535. (10.1525/bio.2013.63.7.6)

[21] Mace GM Collar NJ Gaston KJ Hilton-Taylor C Akçakaya HR Leader-Williams N Milner-Gulland EJ Stuart SN 2008 Quantification of extinction risk: IUCN's system for classifying threatened species. Conserv. Biol. 22, 1424–1442. (10.1111/j.1523-1739.2008.01044.x) 18847444

[22] IUCN Standards and Petitions Subcommittee. 2011 Guidelines for using the IUCN Red List categories and criteria*.* *Version 9.0*.: Prepared by the Standards and Petitions Subcommittee. (http://www.iucnredlist.org/documents/RedListGuidelines.pdf)

[23] Kosmidis K Halley JM Argyrakis P 2005 Language evolution and population dynamics in a system of two interacting species. Physica A 353, 595–612. (10.1016/j.physa.2005.02.038)

[24] Abrams DM Strogatz SH 2003 Linguistics: modelling the dynamics of language death. Nature 424, 900 (10.1038/424900a) 12931177

[25] Lewis MP (ed.) 2009 Ethnologue: languages of the world, 16th edn Dallas, TX: SIL International Online version: http://www.ethnologue.com/

[26] Patriarca M Heinsalu E 2009 Influence of geography on language competition. Physica A 388, 174–186. (10.1016/j.physa.2008.09.034)

[27] Minett JW Wang WS-Y 2008 Modelling endangered languages: the effects of bilingualism and social structure. Lingua 118, 19–45. (10.1016/j.lingua.2007.04.001)

[28] Kandler A 2009 Demography and language competition. Human Biol. 81, 181–210. (10.3378/027.081.0305) 19943743

[29] Nettle D 1998 Explaining global patterns of language diversity. J. Anthropol. Archaeol. 17, 354–374. (10.1006/jaar.1998.0328)

[30] The Catalogue of Endangered Languages at University of Hawaii. 2011 About the Catalogue of the Endangered Languages. See http://www.endangeredlanguages.com/about/.

[31] Balmford A 1996 Extinction filters and current resilience: the significance of past selection pressures for conservation biology. Trends Ecol. Evol. 11, 193–196. (10.1016/0169-5347(96)10026-4) 21237807

[32] Jetz W Rahbek C 2002 Geographic range size and determinants of avian species richness. Science 297, 1548–1551. (10.1126/science.1072779) 12202829

[33] Global Mapping International. 2010 World language mapping system version 16.0. Colorado Springs, CO: Global Mapping International (http://www.worldgeodatasets.com/language/)

[34] Harmon D Loh J 2010 The index of linguistic diversity: a new quantitative measure of trends in the status of the world's languages. Lang. Document. Conserv. 4, 97–151.

[35] Burnham KP Anderson DR 2002 Model selection and multimodel inference: a practical information-theoretic approach, 2nd edn New York, NY: Springer.

[36] Muggeo VMR 2012 segmented: segmented relationships in regression models with breakpoints/changepoints estimation: R package. See: http://cran.r-project.org/web/packages/segmented/index.html.

[37] Bjørnstad ON 2012 *ncf: spatial nonparametric covariance functions*: R package. See: http://cran.r-project.org/web/packages/ncf/index.html.

[38] R Development Core Team. 2012 R: a language and environment for statistical computing. (http://www.R-project.org/). Vienna, Austria: R Foundation for Statistical Computing.

[39] Bivand R 2012 *spdep: spatial dependence: weighting schemes, statistics and models*: R package. See: http://cran.r-project.org/web/packages/spdep/index.html.

[40] Bartoń K 2012 *MuMIn: multi-model inference*: R package. See: http://cran.r-project.org/web/packages/MuMIn/index.html.

[41] Bini LM 2009 Coefficient shifts in geographical ecology: an empirical evaluation of spatial and non-spatial regression. Ecography 32, 193–204. (10.1111/j.1600-0587.2009.05717.x)

[42] Turvey ST Fritz SA 2011 The ghosts of mammals past: biological and geographical patterns of global mammalian extinction across the Holocene. Phil. Trans. R. Soc. B 366, 2564–2576. (10.1098/rstb.2011.0020) 21807737 PMC3138610

[43] Stephens PA Sutherland WJ 1999 Consequences of the Allee effect for behaviour, ecology and conservation. Trends Ecol. Evol. 14, 401–405. (10.1016/S0169-5347(99)01684-5) 10481204

[44] Orme CDL 2006 Global patterns of geographic range size in birds. PLoS Biol. 4, 1276–1283. 10.1371/journal.pbio.0040208 PMC1479698 16774453

[45] Whitton FJS Purvis A Orme CDL Olalla-Tárraga MÁ 2012 Understanding global patterns in amphibian geographic range size: does Rapoport rule? Global Ecol. Biogeogr. 21, 179–190. (10.1111/j.1466-8238.2011.00660.x)

[46] Davies TJ Purvis A Gittleman JL 2009 Quaternary climate change and the geographic ranges of mammals. Am. Nat. 174, 297–307. (10.1086/603614) 19627231

[47] Stevens GC 1989 The latitudinal gradient in geographical range—how so many species coexist in the tropics. Am. Nat. 133, 240–256. (10.1086/284913)

[48] WWF. 2012 Living Planet Report 2012. Gland, Switzerland: WWF International.

[49] Davies RG 2006 Human impacts and the global distribution of extinction risk. Proc. R. Soc. B 273, 2127–2133. (10.1098/rspb.2006.3551) PMC1635517 16901831

[50] Solé RV Corominas-Murtra B Fortuny J 2010 Diversity, competition, extinction: the ecophysics of language change. J. R. Soc. Interface 7, 1647–1664. (10.1098/rsif.2010.0110) 20591847 PMC2988263

[51] Otero-Espinar MV Seoane LF Nieto JJ Mira J 2013 An analytic solution of a model of language competition with bilingualism and interlinguistic similarity. Physica D 264, 17–26. (10.1016/j.physd.2013.08.011)

