# Supplementary material for: Global distribution and drivers of language extinction risk
Source: Proc Biol Sci. 2014 Oct 22;281(1793):20141574. doi: 10.1098/rspb.2014.1574 (PMC4173687; doi:10.1098/rspb.2014.1574)
Supplement: ESM.revised2.Amano [file rspb20141574supp1.doc]

**Electronic Supplementary Material**

**Global distribution and drivers of language extinction risk**

Amano, T.^*^, Sandel, B., Eager, H., Bulteau, E., Svenning, J.-C., Dalsgaard, B., Rahbek, C., Davies, R. G. and Sutherland, W. J.

*To whom correspondence should be addressed. E-mail: amatatsu830@gmail.com

Appendices A, B and C

Supplementary Tables S1-S6

Supplementary Figures S1-S15

**Appendix A.** Justification for using range sizes, speaker population sizes (i.e., numbers of speakers) and speaker growth rates (changes in the number of speakers) as extinction risk components for languages.

The rapid declines in the number of speakers (used in IUCN Criterion A3; see table S3) clearly represent a higher extinction risk, whatever process is involved. Theoretical studies have shown that deterministic declines are always a serious risk, with population size having little effect on time to extinction [1]. Krause [2] and Nettle and Romaine [3] also support the idea, showing examples where even languages with large population size have lost many speakers within a short period of time under steady pressure. The Catalogue of Endangered Languages, another global assessment, also uses speaker number trends as one of the four factors for assessing the vitality levels of languages [4].

Small speaker population sizes (used in Criteria C1 and D1) and small range sizes (Criteria B1 and D2) represent high extinction risk due to demographic and environmental stochasticity, both of which are known as key processes causing the extinction of languages (e.g., environmental stochasticity, such as epidemic outbreaks, tsunami and developments: [3]; demographic stochasticity: [5]). The IUCN criteria B, C and D are based on theoretical studies about the general relationships between population/range sizes and the time to extinction in species under various kinds of environmental and demographic stochasticity [1]. There are certainly some fundamental differences in the extinction processes of species and languages, as represented by bilingualism and revitalisation in languages [2]. However, considering that theoretical studies in linguistics have also shown that the population size, as well as range size, of a language is a key factor in explaining the time to extinction [6], even when accounting for bilingualism [7,8], we believe that these criteria are also effective for assessing the extinction risk of languages. It is also true that minority (small-range/population) languages are more likely to suffer from oppression and adverse language policies [9]. This also seems to be supported by the result of our analysis, which shows that small-range and small-population languages have, in particular, shown speaker declines (figure 1e, f).

Small speaker population sizes have also been used as a criterion to define endangered languages. For example, Derhemi [10] stated that “there is a high probability that with time a neglected minority language will become endangered”. The UNESCO’s assessment of endangered languages [11] also uses the number of speakers as one of the six factors for evaluating language endangerment, stating that, “A small speech community is always at risk”. The same is true in the Catalogue of Endangered Languages [4].

In contrast, small geographical ranges have not been focused on in the assessment of language endangerment so far. However, empirical and theoretical studies suggest that small range sizes will increase the likelihood of extinction through stochasticity both in languages [6] and species [1]. The strong positive correlation between languages’ range and population sizes (figure 1d) also partly supports the effectiveness of using small range size as a measure of high extinction risk in, for example, languages without speaker number estimates. Further, Criterion B1 is not based solely on small range sizes. Instead there must be evidence that the population (i.e., the number of speakers in our study) is in continuing decline or limited to a few locations [12]. Criterion D2 is also not based solely on range size, requiring that the population (with range size smaller than 20km^2^) is prone to the effects of serious threats within a very short time period [12]. Thus, considering that many languages with range sizes smaller than 20km^2^ are also associated with a small number of speakers (median = 2,500) but do not satisfy Criteria A3, B1 and C1 (due to the lack of speaker growth estimates) or D1 (because the number of speakers is, although small, larger than 1000, the threshold to be categorised as Vulnerable under D1), we assumed that all of the languages with range size smaller than 20km^2^ are under such threat and categorised them as Vulnerable. Clearly in the future we need further inspections on potential threats to those languages.

Finally, note that with the range size data used in this study, it is difficult to distinguish if a language originally had a small range size or its range size has contracted recently. However, our primary purpose is to investigate the distribution and drivers of languages’ current extinction risk. Small range and speaker population sizes represent high extinction risks at present, whether it is the language’s original characteristics or a consequence of a recent contraction. Further, based on our analysis (table 1), we can also infer that different processes are at work at different temporal scales to form the current range size and population size of languages. For example, the significant effect of environmental factors on language range and population sizes presumably reflects historical processes determining languages’ original characteristics through diversification and persistence. In contrast, economic growth and globalisation seem to be responsible for more recent speaker declines, mainly since the 1970s (see the time period covered by speaker growth estimates in figure S4). Given the positive, albeit weak, association between small range size and speaker declines (figure 1e), at least some languages in economically-developed regions may have recently contracted their range as well. So, we believe that range contraction has been occurring in economically-developed regions while in other regions with high productivity and heterogeneity, such as the tropics and the Himalayan region, small range sizes have arisen mainly due to environmental features. See **Results** and **Discussion** for more detail.

^1^ Mace, G. M., Collar, N. J., Gaston, K. J., Hilton-Taylor, C., AkÇAkaya, H. R., Leader-Williams, N., Milner-Gulland, E. J. & Stuart, S. N. 2008 Quantification of extinction risk: IUCN's system for classifying threatened species. *Conservation Biology* **22**, 1424-1442.

^2^ Krauss, M. 1992 Endangered languages - the world's languages in crisis. *Language* **68**, 4-10.

^3^ Nettle, D. & Romaine, S. 2000 *Vanishing voices: the extinction of the world's languages*. Oxford, UK: Oxford University Press.

^4^ The Catalogue of Endangered Languages at University of Hawaii. 2011 *About the Catalogue of the Endangered Languages*: Available at: http://www.endangeredlanguages.com/about/.

^5^ Kosmidis, K., Halley, J. M. & Argyrakis, P. 2005 Language evolution and population dynamics in a system of two interacting species. *Physica A: Statistical Mechanics and its Applications* **353**, 595-612.

^6^ Patriarca, M. & Heinsalu, E. 2009 Influence of geography on language competition. *Physica A: Statistical Mechanics and its Applications* **388**, 174-186.

^7^ Minett, J. W. & Wang, W. S.-Y. 2008 Modelling endangered languages: the effects of bilingualism and social structure. *Lingua* **118**, 19-45.

^8^ Kandler, A. 2009 Demography and language competition. *Human Biology* **81**, 181-210.

^9^ Grenoble, L. A. 2011 Language ecology and endangerment. In *The Cambridge Handbook of Endangered Languages* (ed. P. K. Austin & J. Sallabank), pp. 27-44. Cambridge: Cambridge University Press.

^10^ Derhemi, E. 2002 Thematic introduction: protecting endangered minority languages: sociolinguistic perspectives. *International Journal on Multicultural Societies* **4**, 150-161.

^11^ Brenzinger, M., Yamamoto, A., Aikawa, N., Koundiouba, D., Minasyan, A., Dwyer, A., Grinevald, C., Krauss, M., Miyaoka, O., Sakiyama, O., Smeets, R. & Zepeda, O. 2003 *Language vitality and endangerment*. Paris: UNESCO Ad Hoc Expert Group Meeting on Endangered Languages, http://www.unesco.org/culture/en/endangeredlanguages.

^12^ IUCN Standards and Petitions Subcommittee. 2011 *Guidelines for using the IUCN Red List categories and criteria. Version 9.0.*: Prepared by the Standards and Petitions Subcommittee. Downloadable from http://www.iucnredlist.org/documents/RedListGuidelines.pdf.

**Appendix B.** Descriptions of data based on the Atlas of the World’s Languages in Danger and Catalogue of Endangered Languages.

The Atlas of the World’s Languages in Danger [1] provides information on the degrees of endangerment (vulnerable, definitely endangered, severely endangered, critically endangered and extinct) for 2,724 languages, categorised based on the level of intergenerational language transmission [2]. Of the 2,724 languages, we could obtain the geographical range for 2,498 languages from the WLMS database. We then excluded 127 extinct languages, leaving 2,371 languages for the following analysis.

The Catalogue of Endangered Languages lists 3,045 languages, categorised into one of the eight levels of endangerment (at risk, vulnerable, threatened, endangered, severely endangered, critically endangered, dormant and awakening) based on four factors and associated uncertainties [3,4]. We first excluded 84 languages that were categorised as dormant or awakening. Of the remaining languages, we obtained the geographical range for 2,228 languages from the WLMS database, which were then used to map the number of endangered languages for a comparison with that based on the IUCN criteria.

^1^ Moseley, C. 2010 Atlas of the World's Languages in Danger, 3rd edn. Online version: <http://www.unesco.org/culture/en/endangeredlanguages/atlas>. Paris: UNESCO Publishing.

^2^ Moseley, C. 2012 *The UNESCO atlas of the World's languages in danger: context and process*. Cambridge, UK: World Oral Literature Project Occasional Paper 5.

^3^ The Linguist List at Eastern Michigan University and The University of Hawaii at Manoa. 2012 *Endangered Languages*: April 22, 2014. [http://www.endangeredlanguages.com](http://www.endangeredlanguages.com/).

^4^ The Catalogue of Endangered Languages at University of Hawaii. 2011 *About the Catalogue of the Endangered Languages*: Available at: <http://www.endangeredlanguages.com/about/>.

**Appendix C.** Data sources for potential drivers of language extinction risk.

Data on potential drivers of extinction risk were derived from different global data sources: annual mean temperature, annual precipitation, temperature and precipitation seasonality and elevation range (the difference between maximum and minimum elevation for each cell) from the WorldClim [1], vegetation productivity (represented by the Normalized Difference Vegetation Index) from the Global Data Archive 2: AVHRR NDVI 1981 – 2000 (originally from the NASA Goddard DAAC ftp site: http://glcf.umiacs.umd.edu/data/gimms/ and processed by Clark Labs, Clark University), habitat diversity (Shannon diversity index) calculated from the UMD AVHRR Land Cover Classification (http://www.glcf.umd.edu/data/landcover/), mean population density from the Gridded Population of the World v3 (http://sedac.ciesin.columbia.edu/data/collection/gpw-v3), mean population change calculated based on the United Nations World Population Prospect (mean growth between 1980 and 2000: http://esa.un.org/unpd/wpp/Excel-Data/population.htm), Gross Domestic Product (GDP) per capita calculated based on the United Nations National Accounts Main Aggregates Database (mean of GDP per capita in 1980, 1990 and 2000: http://unstats.un.org/unsd/snaama/introduction.asp) and the degree of globalisation calculated as the mean of the KOF Index of Globalisation between 1978 and 2000 [2].

^1^ Hijmans, R. J., Cameron, S. E., Parra, J. L., Jones, P. G. & Jarvis, A. 2005 Very high resolution interpolated climate surfaces for global land areas. *International Journal of Climatology* **25**, 1965-1978.

^2^ Dreher, A. 2006 Does globalization affect growth? Evidence from a new Index of Globalization. *Applied Economics* **38**, 1091-1110.

**Supplementary Tables**

**Table S1.** Hypothesised positive (shown in blue) and negative (red) effects of each driver used as the explanatory variable.

|  | Range size / speaker population size | Speaker growth rate |
| --- | --- | --- |
| **Productivity** |  |  |
| Annual mean temperature  Annual precipitation  Vegetation productivity | Higher productivity allows small groups to be self-sufficient, causing many small languages to evolve and persist [1]. | Higher productivity enhances the growth of speaker populations [1-4]. |
| **Climate variability** |  |  |
| Temperature seasonality  Precipitation seasonality | Stable climate allows small groups to be self-sufficient, causing many small languages to evolve and persist [1,3,5]. | Stable climate allows reliable food production all year round and thus stable growth of speaker populations [1,3]. |
| **Habitat heterogeneity** |  |  |
| Elevation range  Habitat diversity | Elevational and habitat heterogeneities constrain range expansion, causing small range / population size [6]. | Elevational and habitat heterogeneities constrain range expansion and so alleviate the negative effect of other languages [2,3,6,7]. |
| **Socioeconomic factors** |  |  |
| Mean population density | High population density / positive human population growth can directly be linked to an increase in speakers and consequently large range and population size, but can also promote the persistence of small-range and small-population languages through an increase in potential speakers. | High population density / positive human population growth can directly be linked to an increase in speakers [8] but there can be negative impact particularly on minor languages through the growth of a few dominant languages. |
| Mean population change |
| GDP per capita | Modernisation causes the extinction of minor languages and so increases median range / population size [1]. | Modernisation causes declines in the number of speakers in most languages mainly due to language shifts [1]. |
| Globalisation | Globalisation causes the dominance of global languages and so increases median range / population size [9] | Globalisation drives people’s shifts from local languages to a few global languages [9] |
| Language richness |  | Competition with other languages affects the number of speakers negatively in minor and positively in major languages [10,11]. |
| **Land area within the same latitudinal band** | Range size is constrained by the land area with the same climatic conditions [12]. | Culture and technology spread more easily longitudinally, causing a detrimental effect on the persistence of small languages [13]. |

*Although there are other potentially influential drivers of language extinction risk, such as language suppression [14] or adverse language policies [9], the effect of these factors could not be tested due to the lack of relevant information at the global scale.

^1^ Nettle, D. 1998 Explaining global patterns of language diversity. *Journal of Anthropological Archaeology* **17**, 354-374.

^2^ Burnside, W. R., Brown, J. H., Burger, O., Hamilton, M. J., Moses, M. & Bettencourt, L. M. A. 2012 Human macroecology: linking pattern and process in big-picture human ecology. *Biological Reviews* **87**, 194-208.

^3^ Moore, J. L., Manne, L., Brooks, T., Burgess, N. D., Davies, R., Rahbek, C., Williams, P. & Balmford, A. 2002 The distribution of cultural and biological diversity in Africa. *Proceedings of the Royal Society of London Series B-Biological Sciences* **269**, 1645-1653.

^4^ Collard, I. F. & Foley, R. A. 2002 Latitudinal patterns and environmental determinants of recent human cultural diversity: do humans follow biogeographical rules? *Evolutionary Ecology Research* **4**, 371-383.

^5^ Cashdan, E. 2001 Ethnic diversity and its environmental determinants: Effects of climate, pathogens, and habitat diversity. *American Anthropologist* **103**, 968-991.

^6^ Currie, T. E. & Mace, R. 2009 Political complexity predicts the spread of ethnolinguistic groups. *Proceedings of the National Academy of Sciences of the United States of America* **106**, 7339-7344.

^7^ Mace, R. & Pagel, M. 1995 A latitudinal gradient in the density of human languages in North America. *Proceedings of the Royal Society of London Series B-Biological Sciences* **261**, 117-121.

^8^ Graddol, D. 2004 The future of language. *Science* **303**, 1329-1331.

^9^ Grenoble, L. A. 2011 Language ecology and endangerment. In *The Cambridge Handbook of Endangered Languages* (ed. P. K. Austin & J. Sallabank), pp. 27-44. Cambridge: Cambridge University Press.

^10^ Maffi, L. 2005 Linguistic, cultural, and biological diversity. *Annual Review of Anthropology* **34**, 599-617.

^11^ Manne, L. L. 2003 Nothing has yet lasted forever: current and threatened levels of biological and cultural diversity. *Evolutionary Ecology Research* **5**, 517-527.

^12^ Hawkins, B. A. & Diniz-Filho, J. A. F. 2006 Beyond Rapoport's rule: evaluating range size patterns of New World birds in a two-dimensional framework. *Global Ecology and Biogeography* **15**, 461-469.

^13^ Laitin, D. D., Moortgat, J. & Robinson, A. L. 2012 Geographic axes and the persistence of cultural diversity. *Proceedings of the National Academy of Sciences of the United States of America* **109**, 10263-10268.

^14^ Krauss, M. 1992 Endangered languages - the world's languages in crisis. *Language* **68**, 4-10.

**Table S2.** Correlation matrix of 13 potential drivers of language extinction risk. Mean population density, GDP per capita and language richness are log_10_-transformed values. AMT: annual mean temperature, AP: annual precipitation, TS: temperature seasonality, PS: precipitation seasonality, Land band: land area within the same latitudinal band. Combinations with |*r*| > 0.8 are shown in bold.

|  | AMT | AP | TS | PS | Vegetation productivity | Elevation range | Habitat diversity | Mean population density | Population change | GDP per capita | Globalisation  Index | Language richness |
| --- | --- | --- | --- | --- | --- | --- | --- | --- | --- | --- | --- | --- |
| AMT |  |  |  |  |  |  |  |  |  |  |  |  |
| AP | 0.365 |  |  |  |  |  |  |  |  |  |  |  |
| TS | **-0.868** | -0.552 |  |  |  |  |  |  |  |  |  |  |
| PS | 0.331 | -0.230 | -0.180 |  |  |  |  |  |  |  |  |  |
| Vegetation productivity | 0.404 | 0.728 | -0.534 | -0.195 |  |  |  |  |  |  |  |  |
| Elevation range | -0.145 | 0.052 | -0.042 | 0.047 | -0.021 |  |  |  |  |  |  |  |
| Habitat diversity | -0.368 | 0.109 | 0.197 | -0.217 | 0.204 | 0.382 |  |  |  |  |  |  |
| Mean population density | 0.299 | 0.271 | -0.310 | 0.073 | 0.372 | 0.226 | 0.367 |  |  |  |  |  |
| Population change | 0.721 | 0.139 | -0.646 | 0.488 | 0.091 | 0.042 | -0.402 | 0.146 |  |  |  |  |
| GDP per capita | -0.328 | -0.151 | 0.285 | -0.506 | -0.051 | -0.139 | 0.115 | -0.358 | -0.434 |  |  |  |
| Globalisation index | -0.343 | -0.082 | 0.255 | -0.508 | 0.045 | -0.171 | 0.159 | -0.374 | -0.455 | **0.938** |  |  |
| Language richness | 0.333 | 0.455 | -0.408 | 0.141 | 0.353 | 0.220 | 0.110 | 0.333 | 0.327 | -0.407 | -0.323 |  |
| Land band | **-0.804** | -0.383 | **0.841** | -0.253 | -0.429 | -0.003 | 0.249 | -0.160 | -0.635 | 0.295 | 0.278 | -0.278 |

**Table S3.** Summary of the five IUCN criteria used to evaluate if a language belongs in a threatened category (Critically Endangered, Endangered or Vulnerable).

| Criteria | Definition | Data used | Critically Endangered | Endangered | Vulnerable |
| --- | --- | --- | --- | --- | --- |
| IUCN A3 | Population reduction projected based on an index of abundance | Population declines projected using the estimated growth rates over three generations (3 × 30 years) | < 0.982 (> 80% decline) | < 0.992 (> 50% decline) | < 0.996 (> 30% decline) |
| IUCN B1 | Extent of occurrence +  number of locations +  continuing decline in the number of mature individuals | Range size | < 100 km^2^ | < 5,000 km^2^ | < 20,000 km^2^ |
|  |  | Number of polygons in the WLMS database | 1 |  5 | ≤ 10 |
|  |  | Population declines based on the estimated growth rates | < 1.000 | < 1.000 | < 1.000 |
| IUCN C1 | Number of mature individuals + continuing decline | Population size | < 250 | < 2,500 | < 10,000 |
|  |  | Population declines projected using the estimated growth rates | < 0.991 (> 25% decline over one generation) | < 0.996 (> 20% decline over 2 generations) | < 0.999 (> 10% decline over 3 generations) |
| IUCN D1 | Number of mature individuals | Population size | < 50 | < 250 | < 1,000 |
| IUCN D2 | Restricted area of occurrence (the population is prone to threats within a very short time period) | Range size |  |  | < 20km^2^ |

**Table S4.** Akaike Information Criterion (AIC) of the null, linear, quadratic and segmented regression models for the relationship between range size and population size, speaker growth rate and range size, and speaker growth rate and population size of languages. Note that the initial population size (i.e., the record of speaker population size in the oldest year) was used in the analysis of the relationship between speaker growth rate and population size, in order to avoid circularity. The model with the smallest AIC is shown in bold. *Δ*AIC represents a difference in AIC from the model with the smallest AIC. Adjusted *R^2^* for each model is also shown. Since we did not have sufficient information on the phylogeny of all the languages, we did not account for potential phylogenetic non-independence of model residuals in this analysis. However, there was a huge (> 25) difference in AIC between the best and second best models for the relationships between range size and population size and speaker growth rate and population size, so the conclusion that segmented regression is the single best model seems robust, at least for the two relationships above, irrespective of potential phylogenetic autocorrelation.

|  | Range size vs. population size  (*n* = 6,708) | | | Speaker growth rate vs. range size  (*n* = 613) | | | Speaker growth rate vs. population size  (*n* = 649) | | |
| --- | --- | --- | --- | --- | --- | --- | --- | --- | --- |
|  | AIC | *Δ*AIC | Adjusted *R^2^* | AIC | *Δ*AIC | Adjusted *R^2^* | AIC | *Δ*AIC | Adjusted *R^2^* |
| Null model: ~ *a* | 18216.86 | 3413.57 |  | -2050.21 | 5.25 |  | -1079.695 | 119.118 |  |
| Linear model: ~ *a* + *bx* | 15424.63 | 621.34 | 0.331 | -2053.20 | 2.26 | 0.006 | -1113.475 | 85.338 | 0.052 |
| Quadratic model: ~ *a* + *bx* + *cx^2^* | 14837.28 | 33.99 | 0.391 | -2054.11 | 1.35 | 0.010 | -1172.465 | 26.348 | 0.136 |
| Segmented regression  ~ *a* + *b_1_x* for *x* < *ψ*  ~ *a* + *b_1_x* + *b_2_*(*x* - *ψ*) for *x* ≥ *ψ* | **14803.29** | **0** | **0.394** | **-2055.46** | **0** | **0.013** | **-1198.813** | **0** | **0.172** |

**Table S5.** Model-averaged results based on simultaneous autoregressive error models for the effect of environmental and socioeconomic factors on risk due to insufficient language intergenerational transmission. Coefficients, their SEs and *z* values show weighted-average values across 95% sets of simultaneous autoregressive error models with different parameter combinations by Akaike weights (*w_i_*). Σ *w_i_* for each variable shows the sum of *w_i_* of models including the variable, reflecting the relative importance of each variable. Results with *z* values > 2.0 are shown in bold. Nagelkerke pseudo-*R^2^* for the full model was 0.63.

| Explanatory variables | Coefficients | SE | *z* values | Σ *w_i_* |
| --- | --- | --- | --- | --- |
| Environmental factors |  |  |  |  |
| Annual precipitation | 0.5510^-5^ | 1.4310^-5^ | 0.39 | 0.27 |
| Vegetation productivity | -6.9710^-4^ | 4.2610^-4^ | 1.64 | 0.58 |
| Temperature seasonality | **2.0210^-5^** | **0.3310^-5^** | **6.10** | **1.00** |
| Precipitation seasonality | -1.9010^-4^ | 3.0010^-4^ | 0.64 | 0.30 |
| Elevation range | 0.1110^-6^ | 5.3410^-6^ | 0.02 | 0.25 |
| Habitat diversity | 0.3710^-2^ | 1.1810^-2^ | 0.32 | 0.26 |
| Socioeconomic factors |  |  |  |  |
| Mean population density | **-2.1310^-2^** | **0.6710^-2^** | **3.19** | **1.00** |
| Population change | -1.2410^-2^ | 1.3710^-2^ | 0.90 | 0.34 |
| GDP per capita | **1.1810^-1^** | **0.2010^-1^** | **5.89** | **1.00** |
| Language richness | **2.8410^-1^** | **0.1510^-1^** | **18.49** | **1.00** |

**Table S6.** Model-averaged results based on ordinary least squares models for the effect of environmental and socioeconomic factors on language range size, population size and speaker growth rate. Language richness was not included in the models for range size and speaker population size. Coefficients, their SEs and *z* values show weighted-average values across 95% sets of ordinary least squares models with different parameter combinations by Akaike weights (*w_i_*). Σ *w_i_* for each variable shows the sum of *w_i_* of models including the variable, reflecting the relative importance of each variable. Results with *z* values > 2.0 are shown in bold. *R^2^* for the full model was 0.25, 0.22 and 0.10 for range size, speaker population size and speaker growth rate, respectively.

| Explanatory variables | Geographical range size | | | |  | Speaker population size | | | |  | Speaker growth rate | | | |
| --- | --- | --- | --- | --- | --- | --- | --- | --- | --- | --- | --- | --- | --- | --- |
|  | Coefficients | SE | *z* | Σ *w_i_* |  | Coefficients | SE | *z* values | Σ *w_i_* |  | Coefficients | SE | *z* values | Σ *w_i_* |
| Environmental factors |  |  |  |  |  |  |  |  |  |  |  |  |  |  |
| Annual precipitation | **-6.6010^-4^** | **0.4310^-4^** | **15.28** | **1.00** |  | **-7.8410^-4^** | **0.5110^-4^** | **15.24** | **1.00** |  | 1.3810^-6^ | 2.1410^-6^ | 0.64 | 0.32 |
| Vegetation productivity | **5.2410^-3^** | **1.4510^-3^** | **3.62** | **1.00** |  | 0.5210^-3^ | 2.1310^-3^ | 0.24 | 0.23 |  | 2.5110^-5^ | 7.2210^-5^ | 0.35 | 0.28 |
| Temperature seasonality | 3.3010^-6^ | 6.5610^-6^ | 0.51 | 0.18 |  | 3.6910^-6^ | 9.6310^-6^ | 0.38 | 0.26 |  | **-2.8710^-6^** | **0.3110^-6^** | **9.24** | **1.00** |
| Precipitation seasonality | -9.9210^-4^ | 7.2410^-4^ | 1.37 | 0.40 |  | **-5.1310^-3^** | **1.0410^-3^** | **4.94** | **1.00** |  | **-1.0310^-4^** | **0.4010^-4^** | **2.56** | **0.90** |
| Elevation range | **-1.3510^-4^** | **0.2010^-4^** | **6.78** | **1.00** |  | **-1.7310^-4^** | **0.2710^-4^** | **6.36** | **1.00** |  | 1.5610^-6^ | 1.1010^-6^ | 1.41 | 0.51 |
| Habitat diversity | **-0.23** | **0.05** | **4.80** | **1.00** |  | -0.04 | 0.07 | 0.51 | 0.27 |  | **1.5210^-2^** | **0.2710^-2^** | **5.65** | **1.00** |
| Socioeconomic factors |  |  |  |  |  |  |  |  |  |  |  |  |  |  |
| Mean population density | **0.10** | **0.02** | **4.99** | **1.00** |  | **0.63** | **0.03** | **22.81** | **1.00** |  | **-3.2210^-3^** | **1.1210^-3^** | **2.88** | **0.98** |
| Population change | **-0.37** | **0.03** | **13.59** | **1.00** |  | **-0.33** | **0.04** | **8.30** | **1.00** |  | -0.3310^-3^ | 1.8610^-3^ | 0.18 | 0.26 |
| GDP per capita | **0.19** | **0.04** | **5.01** | **1.00** |  | 0.05 | 0.06 | 0.81 | 0.31 |  | **-6.0610^-3^** | **2.2410^-3^** | **2.71** | **0.95** |
| Language richness |  |  |  |  |  |  |  |  |  |  | 3.5810^-3^ | 3.3710^-3^ | 1.06 | 0.40 |

**Supplementary Figures**

**Figure S1.** Comparison of the global maps of (a) total language richness and (b) the richness of languages with speaker growth estimates.

**Figure S2.** Relationship between total language richness and the richness of languages with speaker growth estimates.

**Figure S3.** Histograms of (a) geographical range size and (b) initial population size (the record of speaker population size in the oldest survey year) in 649 languages with speaker growth estimates (orange bars), 1500 languages in the ILD Database (blue) and all languages in the WLMS database (grey). Mean values are shown with red (649 languages), blue (1500 languages) and black (all languages) lines. In (a), the y-axis on the left is for all languages in the WLMS database and the right one is for both the 649 and 1500 languages. Note that the initial population size was only available for languages in the ILD Database. Range size and initial population size were significantly larger in the 649 languages with speaker growth estimates than in the 1,500 languages in the ILD Database (range size: *t* = -5.41, *df* = 1875, *p* < 0.0001, initial population size: *t* = -3.58, *df* = 1934, *p* = 0.0003) and all available languages in the WLMS database (range size: *t* = -6.76, *df* = 6970, *p* < 0.0001). However, the biases were very small and the shape and range of the histograms were quite similar with each other.

**Figure S4.** The survey period covered by the Index of Linguistic Diversity Database for 649 languages used in the analysis. Each line connects years of the first and last records for each language.

**Figure S5.** Moran's *I* correlograms of residuals in ordinary least squares models (black) and simultaneous autoregressive (SAR) error models (red) for the analysis of (a) range size, (b) population size, (c) speaker growth rate and (d) intergenerational transmission of languages. Distance classes were defined at 200-km intervals, ranging from 200 - 5000 km. SAR error models were fitted using the neighborhood distances with the smallest AIC (range size: 450 km, population size: 350 km, speaker growth rate: 300 km and intergenerational transmission: 350 km; see **Materials and methods** for more detail).

**Figure S6.** The latitudinal gradient in median (a) range size (km^2^), (b) population size and (c) speaker growth rate of languages. The solid red lines show latitudinal medians and the grey points show median values for each 2° grid cell. Medians were calculated for log_10_-transformed range size and speaker population size and for speaker growth rate.

**Figure S7.** The global map of language extinction risk due to insufficient intergenerational transmission, calculated as the median value of all languages within each cell, assuming vulnerable = 1, definitely endangered = 2, severely endangered = 3, critically endangered = 4 and all others = 0 (see **Material and methods** in the main text and Appendix B for more detail).

**Figure S8.** Partial residual plots of the relationship between language range size and its drivers: (a) vegetation productivity, (b) elevation range, (c) habitat diversity, (d) population change and (e) GDP per capita. The plots show the individual effects of each variable after effects of other variables and spatial autocorrelation have been partialled out. Variables shown here are those selected in the best simultaneous autoregressive error model (i.e., that with the smallest AIC) and lines represent regression lines based on coefficients estimated in the best model. Another important driver, annual precipitation, is shown in figure 2d.

**Figure S9.** Partial residual plots of the relationship between language population size and its drivers: (a) elevation range, (b) mean population density and (c) population change. The plots show the individual effects of each variable after effects of other variables and spatial autocorrelation have been partialled out. Variables shown here are those selected in the best simultaneous autoregressive error model (i.e., that with the smallest AIC) and lines represent regression lines based on coefficients estimated in the best model. Another important driver, annual precipitation, is shown in figure 2e.

**Figure S10.** Partial residual plot of the relationship between language speaker growth rate and its driver, temperature seasonality. The plot shows the individual effect of temperature seasonality after effects of other variables and spatial autocorrelation have been partialled out. Temperature seasonality was selected in the best simultaneous autoregressive error model (i.e., that with the smallest AIC) and the line represents a regression line based on the coefficient estimated in the best model. Another important driver, GDP per capita, is shown in figure 2f.

**Figure S11.** Partial residual plots of the relationship between risk due to insufficient intergenerational language transmission and its drivers: (a) GDP per capita, (b) temperature seasonality, (c) mean population density and (d) language richness. The plots show the individual effects of each variable after effects of other variables and spatial autocorrelation have been partialled out. Variables shown here are those with *z* values > 2.0 (see Table S5) and lines represent regression lines based on coefficients estimated in the best simultaneous autoregressive error model (i.e., that with the smallest AIC).

**Figure S12.** Nine explanatory variables used in the analysis of range size, population size, speaker growth rate and intergenerational transmission of languages. Language richness was also used and shown in figure S1a. Population density and GDP per capita are log_10_-transformed.

**Figure S13.** Global maps of threatened language richness categorised using (a) IUCN D2 (based only on range size), (b) D1 (speaker population size), (c) A3 (speaker declines), (d) B1 (range size and speaker declines) and (e) C1 (speaker population size and speaker declines).

**Figure S14.** The global map of threatened language richness based on the Catalogue of Endangered Languages.

**Figure S15.** Relationship between threatened language richness based on the IUCN criteria and that based on the Catalogue of Endangered Languages. Spearman’s rank correlation coefficient was 0.57.
